# Use of Fertility Awareness-Based Methods for Pregnancy Prevention Among Ghanaian Women: A Nationally Representative Cross-Sectional Survey

**DOI:** 10.9745/GHSP-D-20-00601

**Published:** 2021-06-30

**Authors:** Chelsea B. Polis, Easmon Otupiri, Suzanne O. Bell, Roderick Larsen-Reindorf

**Affiliations:** a Guttmacher Institute, New York, New York, USA.; b Johns Hopkins Bloomberg School of Public Health, Baltimore, Maryland, USA.; c School of Public Health, Kwame Nkrumah University of Science and Technology, Ghana.

## Abstract

Few studies in low- and middle-income countries have examined the use of fertility awareness-based methods (FABMs) for pregnancy prevention. Understanding the prevalence of FABM use among Ghanaian contraceptors and the characteristics and practices of users is essential. Our 2018 nationally representative survey of Ghanaian women included detailed questions on the use of rhythm and Standard Days Method/Cycle Beads (SDM). After considering multimethod use patterns, we estimated likely FABM prevalence among contraceptors, identified characteristics associated with current use of an FABM (vs. current use of a hormonal method/intrauterine device [IUD]), and described how women report using FABMs. At least 18% of contracepting Ghanaian women likely use an FABM, though this may be underreported. Among FABM users, 57% reported current use of an FABM alone; the remainder reported concurrent use of other methods. Women who were older, richer, more educated, and had fewer children had higher odds of current FABM use versus IUD/hormonal method. Although FABM users were more likely than other contraceptors to correctly identify the approximate fertile time, only 50% of FABM users did so correctly. Most (92%) rhythm users were interested in making their method use more effective. While 72% had heard of SDM, less than 25% had heard of various other ways to make the rhythm method more effective. Only 17% of rhythm users had ever discussed the method with a health professional. Rhythm users indicated substantial willingness to track additional biomarkers (e.g., daily temperature or cervical mucus) or to use a phone to enhance the effectiveness of their method, and most indicated no substantial difficulty getting partners to abstain or withdraw on fertile days. A nontrivial proportion of reproductive age Ghanaian women are using an FABM, nearly all of whom are interested in learning how to improve its effectiveness. The family planning field should better address these women's contraceptive needs in commitment to reproductive autonomy and choice.

## Abstract (precis)

At least 18% of Ghanaian female contraceptors rely primarily upon a fertility awareness-based method (FABM), and most wish to learn how to improve its effectiveness but are insufficiently supported to do so. Researchers, programmers, and funders should better understand and address FABM users' needs, in commitment to reproductive autonomy and choice.

## Teaser

**Key Findings:**

- Standard approaches underestimate fertility awareness-based method (FABM) use; we posit that at least 18% of contracepting Ghanaian women likely use an FABM.
- Economically and educationally advantaged women had higher odds of current FABM use (versus intrauterine device/hormonal method), which may suggest deliberateness of method selection (as opposed to limited access to alternatives).
- Most (92%) rhythm users wish to improve the effectiveness of their method, and many are willing to track additional biomarkers, but only 17% ever discussed their method with a health professional.

## INTRODUCTION

Understanding the extent of people's reliance on fertility awareness-based contraceptive methods (FABMs) and the characteristics and practices of such users is essential to comprehensively meeting reproductive needs. A concise introduction to FABMs can be found elsewhere.^1^ In brief, FABMs aim to identify the span of days during each menstrual cycle when sexual intercourse is most likely to result in pregnancy (the “fertile window”). Depending on the requirements of the specific FABM, users track changes in 1 or more fertility biomarkers (e.g., menstrual start dates, basal body temperatures, cervical mucus or cervical position, and urinary hormone metabolites) in attempts to identify their fertile window during each menstrual cycle. Certain FABMs can be used to either plan or prevent pregnancy. If pregnancy prevention is desired, users can either avoid penile-vaginal intercourse or use other methods (e.g., barrier methods, withdrawal, etc.) on days the method identifies as fertile.

Such FABM users are taking action to avoid conceiving but may be more vulnerable to unintended pregnancy compared with users of certain other contraceptive methods. For example, in low-income countries, an estimated 19% of those who report using the rhythm method (a type of FABM, described in greater detail below) will experience an unintended pregnancy in the first year of use, compared with 0.3% of implant users.^2^ Contraceptive effectiveness is a key attribute for many people choosing a method, but other method characteristics influence contraceptive decision making, such as safety, side effects, impact on bleeding patterns, cost, ease of use, protection from sexually transmitted infections, privacy, etc.^3^^–^^7^ Despite a common assumption that people resort to less effective contraceptive methods due to a lack of access to more effective options, these choices often reflect user preferences.^8^^–^^13^ For example, side effects and health concerns are the most common reasons for nonuse or discontinuation of hormonal contraception among women in low-income countries who do not desire pregnancy,^14^^,^^15^ whereas methods perceived as “natural” may appeal to people with these concerns.^12^

Despite a common assumption that people resort to less effective contraceptive methods due to a lack of access to more effective options, these choices often reflect user preferences.

FABMs have received relatively limited attention in the contraceptive literature. Rhythm is perhaps the most widely known FABM, although many who report using rhythm are not likely using its formal requirements.^16^ Relatively little is known about how those who identify as rhythm users actually use the method and the extent to which they rely on other contraceptive methods during the fertile window versus abstaining from sex during this period (i.e., practice periodic abstinence). Rhythm is typically classified as a traditional method. However, certain other FABMs (e.g., Standard Days Method [SDM], TwoDay Method, etc.) are often classified as modern—although these classifications are not universal (e.g., the United Nations Population Division does not classify these methods as modern)—and the nomenclature is imperfect.^17^^–^^21^ In addition to FABMs, some people use menstrual tracking smartphone apps or devices to time sexual intercourse for pregnancy prevention. Although 2 apps have received clearance from the United States Food and Drug Administration for use as a contraceptive method (Natural Cycles in 2018,^22^ which also received CE Marking in Europe, and Clue in 2021^23^), most such apps or devices are not tested or indicated for this purpose and may offer predictions of unknown accuracy regarding the timing of fertile days.^24^^,^^25^

Research focused specifically on FABM use in low- and middle-income countries (LMICs) has been particularly limited (with exceptions from the United States Agency for International Development and the Institute for Reproductive Health at Georgetown University^18^^,^^26^^–^^31^). However, there are existing and emerging reasons for greater understanding around FABM use in LMICs. First, our understanding of contraceptive decision making is incomplete without understanding people who select contraceptive options that are not among the most highly effective. Understanding such choices can help to support client-centered approaches to contraceptive counseling, programming, and method development. Second, FABMs use is increasing in some contexts,^32^ and multiple researchers have identified substantial underestimation of methods (including rhythm) in nationally representative surveys.^12^^,^^13^^,^^33^^–^^35^ This suggests incomplete existing information on the prevalence of FABM use, which impacts our understanding of who chooses FABMs and impacts estimation of other key metrics (e.g., unmet need for contraception). Third, without understanding how people employ these methods, we have little ability to identify approaches that enhance the effectiveness of their contraceptive practices while respecting their contraceptive preferences.

Our understanding of contraceptive decision making is incomplete without understanding people who select contraceptive options that are not among the most highly effective.

Additional reasons for understanding FABM use in LMICs have emerged more recently. First, the coronavirus disease (COVID-19) pandemic has limited access to contraceptive services, prompting calls by some international organizations for counseling on FABMs,^36^ which are less vulnerable than other methods to commodity supply chain disruptions.^37^ Furthermore, there is an increasing understanding of the application of principles of self-care to sexual and reproductive health worldwide,^38^ as well as emerging literature on whether fertility knowledge (e.g., regarding the timing of ovulation or impacts of age on fertility) impacts reproductive outcomes.^39^^,^^40^ Although FABMs are not appropriate for all individuals, they can assist certain couples to avoid (or seek) pregnancy with limited or no clinical support after being trained in and correctly using the method. Finally, the increasing development of new technologies will continue to provide new tools that facilitate use of FABMs and may increase their prevalence. The contraceptive field can play a useful role in helping people who prefer a method they may perceive as “natural” in understanding if new technologies have reliable evidence on contraceptive effectiveness^41^ and to optimize the effectiveness of chosen methods.

To expand understanding of FABM use in LMICs, we aimed to examine the use of 2 FABMs (rhythm method and SDM/CycleBeads) in a nationally representative sample of Ghanaian women. Our objectives were to estimate the prevalence of FABM use among Ghanaian contraceptors (including careful consideration of multimethod use patterns), understand characteristics associated with choosing an FABM (relative to choosing an intrauterine device [IUD] or hormonal method), and collect detailed information on various aspects of how Ghanaian women use FABMs. This article also aims to contribute to improvements in measuring FABM use across diverse contexts by detailing methodological considerations around terminology and definitions used for FABMs in large-scale surveys and how these might be improved.

We aimed to estimate the prevalence of FABM use among Ghanaian contraceptors, understand characteristics associated with choosing an FABM, and collect information on how Ghanaian women use FABMs.

## METHODS

### Survey Design and Implementation

Our survey design and sampling have been described in detail elsewhere.^42^ Briefly, data for this analysis were drawn from a nationally representative, community-based survey of reproductive-aged (15–49 years) women in Ghana (N=4,722), conducted in 2018 as part of a larger study on abortion incidence in Ghana.^43^ The survey was a collaborative effort between the Guttmacher Institute, the Kwame Nkrumah University of Science and Technology (KNUST), and the Performance Monitoring and Accountability 2020 team at the Johns Hopkins Bloomberg School of Public Health. We used a multistage stratified cluster sampling design and probability-proportional-to-size sampling to select 100 enumeration areas, each consisting of approximately 200 households. We then listed, mapped, and randomly selected 42 households in each selected enumeration area. In those households, we conducted a short household survey to collect socioeconomic information and to identify eligible female respondents. We invited all eligible women to give informed consent and participate in the full survey. Trained resident enumerators collected data for both surveys in a private area, using an Android smartphone enabled with Open Data Kit electronic data collection software. Households with one or more participating respondents who completed the full survey received a bar of soap. The Institutional Review Boards of the Guttmacher Institute, the KNUST Committee on Human Research, Publication and Ethics, and the Johns Hopkins Bloomberg School of Public Health provided ethical approval. Prior analyses of these data showed that among all respondents aged 15–49 years in our sample who had ever had sex (n=4,139), 33.7% currently reported using any contraceptive method.^44^

### Identifying Rhythm or SDM Users

Traditional methods and modern FABMs are likely underreported in many surveys, including Demographic and Health Surveys (DHS). Studies suggest that some respondents interpret filtering questions (e.g., “are you or your partner currently doing something or using any method to delay or avoid getting pregnant?”) as pertaining only to modern or non-“natural” methods.^12^^,^^13^^,^^33^^,^^34^ Additionally, when asked to spontaneously name methods used, a qualitative study of 48 Ghanaian women found that “fertility awareness methods were rarely spontaneously mentioned as a way to prevent pregnancy…yet counting days was almost universally used at one time or another and participants described it as a taken-for-granted part of a woman's life.”^34^ Other studies suggest that women using multiple methods (e.g., an FABM with condoms during the fertile window) may potentially report only condom use,^12^ unless, for example, she is asked about the use of all potential methods.

Our study did not address the first concern as we did use the filtering question, but it did address the second concern by asking about the use of each method among those who affirmed using a method. Specifically, survey respondents were asked, “Have you or a partner ever done something or used any method to delay or avoid getting pregnant?” We asked respondents who said “yes” to spontaneously list methods ever used, and then the interviewer asked about each of the possible methods to capture users who self-identified as using any of these methods (female and male sterilization, implants, IUDs, injectables (3 month and 1 month), pill, emergency contraception (EC), male and female condoms, diaphragm, foam or jelly, SDM/Cycle Beads, lactational amenorrhea method, N-tablet, rhythm method, withdrawal, washing, or “other traditional method”). The interviewer next followed a similar process for current method use by asking nonpregnant women, “Are you or your partner currently doing something or using any method to delay or avoid getting pregnant?” Again, we only asked respondents who said “yes” to list methods currently used, before being asked about each possible method, to self-identify as current users.

Like DHS and other surveys, our survey may suffer from underreporting of traditional or “natural” methods, if respondents answered “no” to both filtering questions under the assumption that traditional or “natural” methods do not count. Therefore, we interpret our prevalence estimates as a lower bound. However, each respondent who acknowledged ever or currently doing something or using any method to delay or avoid pregnancy was probed on all possible contraceptive methods.

### Data Analysis

Method-specific prevalence is typically calculated based upon the most effective method a person reports currently using. For example, each DHS survey contains a hierarchical list of methods, ordered by typical use contraceptive effectiveness estimates. If a DHS respondent reports currently using both SDM and condoms, she is considered a condom user in prevalence estimation. This approach may underestimate FABM prevalence, as some FABM users use barrier methods or EC during the fertile window^34^^,^^45^ and would thus be coded as a barrier or EC user, despite having sex with no contraception on days that the method (correctly or incorrectly) identifies as infertile.

Method-specific prevalence is typically calculated based on the most effective method a person reports currently using, but this approach may underestimate FABM prevalence.

To address this underestimation issue, we used an approach previously applied to data from the United States^46^ to estimate likely FABM prevalence among Ghanaian contraceptors. First, we calculated a standard prevalence estimate based on “most effective” method (using the ordering represented in Table 1); as already noted, this approach may underestimate FABM use. Next, we recalculated prevalence, incorporating information on multiple method use. That is, a person who reported currently using 2 or more methods would be included in all reported method categories for method-specific prevalence estimation. These are likely overestimates since some women use an FABM with a highly effective method (such as sterilization, an IUD, or hormonal contraception), which likely serves as their primary mode of contraception. Therefore, to identify the most likely FABM prevalence estimate, it is necessary to examine specific patterns of multiple method use among FABM users. We classified respondents who reported any current use of an FABM into 3 categories:

- Category A: Reported current use of an FABM alone or with current use of a less effective method (i.e., withdrawal, washing, etc.)
- Category B: Reported current use of an FABM in conjunction with current use of barrier methods and/or EC
- Category C: Reported current use of an FABM in conjunction with current use of a highly effective method (i.e., hormonal contraception, an IUD, or sterilization).

**TABLE 1.** Percent Distribution of Contraceptors^a^ Aged 15–49, by Current Contraceptive Method(s) Used at Interview, Ghana, 2018

	Among Contraceptors (Unweighted n=1,165),^a^ by Most Effective Method Reported Weighted % and 95% CI	Among Contraceptors (Unweighted n=1,165),^a^ By All Methods Reported Weighted % and 95% CI^b^
**Female sterilization**	3.5 (2.4, 5.0)	3.5 (2.4, 5.0)
**Hormonal methods**		
Implant	22.9 (19.6, 26.7)	22.9 (19.6, 26.7)
Intrauterine device	2.5 (1.6, 4.0)	2.6 (1.6, 4.1)
3-month injectables	19.2 (16.7, 22.0)	20.4 (17.8, 23.3)
1-month injectables	1.7 (1.1, 2.7)	2.0 (1.3, 3.1)
Pill	12.3 (10.0, 15.0)	13.8 (11.3, 16.8)
Emergency contraception	7.5 (5.7, 9.7)	8.2 (6.3, 10.4)
**Condoms**		
Male condom	7.8 (6.1, 9.8)	11.8 (9.6, 14.3)
Female condom	0.1 (0.0, 1.1)	0.3 (0.1, 1.0)
**Traditional methods and FABMs**		
Standard Days Method	4.3 (2.5, 7.1)	6.0 (3.7, 9.4)
Lactational Amenorrhea Method	0.9 (0.5, 1.8)	1.0 (0.5, 1.9)
Rhythm	9.2 (6.9, 12.3)	13.6 (10.5, 17.3)
Withdrawal	3.4 (2.2, 5.1)	12.5 (9.7, 15.9)
N-Tablet^c^	2.1 (1.1, 4.0)	3.6 (2.1, 5.9)
Washing	0.3 (0.0, 0.9)	2.4 (1.3, 4.4)
Other traditional methods (unspecified)	2.3 (1.4, 3.6)	3.0 (2.1, 4.5)

Abbreviations: CI, confidence interval; FABM, fertility awareness-based method.

a Denominator includes women who reported currently using contraception, being sexually experienced, not being currently pregnant, and not currently trying for pregnancy.

b Indicates percent of all contraceptive users that reported using this method at the time of interview, either individually or as part of multiple method use.

Percentages do not sum to 100% because more than one method may have been used at the time of interview. Respondents could list as many methods as necessary.

c N-Tablet refers to Primolut N, a norethisterone pill approved for treating menstrual disorders, which women in Ghana report using as a peri-coital contraceptive method, despite a lack of evidence that it is effective or safe for contraceptive use.

Note: No participants reported that they or their partner was currently using male sterilization, diaphragm, or foam/jelly.

As in a prior study,^46^ we considered women in Category A as definite FABM users, women in Category B as likely FABM users, and women in Category C as relying primarily on a highly effective (non-FABM) method. Thus, we included women in Categories A or B in our FABM prevalence estimation.

We calculated prevalence estimates among “contraceptors,”* excluding women who: did not report that they (or their partner) were “currently doing something or using any method to delay or avoid getting pregnant”; women who were currently trying for pregnancy, women who were currently pregnant, and women who reported never having had sexual intercourse. We considered the remaining 1,165 participants “contraceptors.”

Next, we conducted bivariate and multivariable logistic regressions to identify characteristics we hypothesized to be associated with being a current (definite or likely) FABM user versus a current user of hormonal contraceptives or an IUD, and that were available in these data. Characteristics we examined included ecological zone (Northern, Middle, Central), residence (urban vs. rural), age (continuous), marital status (currently married/cohabiting, formerly married or cohabiting, never married or cohabiting), parity (continuous), education (none, primary or middle, secondary), religion (none, any Christian, Muslim, traditional/other), wealth (poorest 60% vs. richest 40%), importance of avoiding a pregnancy now (very important, somewhat important, not at all important), and correct knowledge of approximate fertility time (incorrect vs. correct). We present a multivariable model including all variables hypothesized to be relevant (a sensitivity analysis demonstrated similar results when including only those variables significant at *P*≤.05). Finally, we calculated descriptive statistics on a series of detailed questions asking FABM users about their knowledge of and ways in which they practice FABMs.

We performed analysis in Stata version 16, accounting for the complex survey design using survey weights to adjust for the probability of selection and the Taylor Linearization method to calculate standard errors that correctly account for clustering. We also used the *subpop* option to restrict to appropriate analytic populations.

## RESULTS

### FABM Prevalence Among Contraceptors

Among contraceptors, 9.2% reported rhythm and 4.3% reported SDM as their most effective method (Table 1). As previously noted, these are likely underestimates. Calculating prevalence among contraceptors based on all methods reported increased rhythm prevalence to 13.6% and SDM prevalence to 6% (Table 1). As mentioned, these are likely overestimates, so we examined specific patterns of multimethod use among FABM users.

Our data revealed a diverse range of FABM method use patterns. Among all women who reported currently using rhythm or SDM, over half (57.3%) reported using that FABM without any additional methods (Table 2). The next most common pattern of multimethod use among rhythm users involved either EC or withdrawal (among 5.9% and 5.6% of women who reported rhythm use, respectively), and among SDM users, involved either withdrawal or male condoms (among 11.9% and 8.6% of women who reported SDM, respectively). If classified by most effective method, 68% and 72% of women who reported currently using rhythm or SDM, respectively, would be classified as a user of the FABM they reported (Table 2, category A). Approximately 23% and 28% of women who reported using rhythm or SDM, respectively, would be classified as either a user of male condoms or EC using the standard prevalence estimation approach; though these women may rely primarily on an FABM and use condoms and/or EC episodically during their presumed fertile window (Table 2, category B). Finally, 9% of women who reported using rhythm (and 0% of women who reported using SDM), reported also currently using a permanent, long-acting reversible, or hormonal method, and would be classified as a user of that more highly effective method using the standard prevalence estimation approach (Table 2, category C).

Our data revealed a diverse range of FABM method use patterns.

**TABLE 2.** Use Patterns Among Women Reporting Current Use of Rhythm or SDM, With or Without Current Use of Other Methods

Rhythm	1. Reported Current Use of Rhythm Plus Other Method	Weighted % Among Those Reporting Rhythm (Unweighted n/N)	2. Classification Using “Most Effective Method” Approach	3. Among Women Who Reported Rhythm^a^ Weighted % (Unweighted n/N)
A. Relies primarily on rhythm	+ No other method	57.3% (89/156)	A rhythm user	68% (106/156)
	+ Withdrawal	5.6% (9/156)		
	+ Withdrawal + washing	3.4% (5/156)		
	+ N tablet	1.3% (2/156)		
	+ Washing	<1% (1/156)		
B. May rely primarily on rhythm and periodic use of EC and/or condoms^b^	+ EC	5.9% (7/156)	A male condom or EC user	23% (36/156)
	+ Male condoms + withdrawal	5.2% (8/156)		
	+ EC + male condoms + withdrawal	1% (2/156)		
	+ Male condoms	1% (2/156)		
	+ Other unique combinations	9.9% (17/156)^c^		
C. Uses rhythm but relies primarily on a modern method	+ 3 monthly injectables	1.7% (3/156)	A user of a permanent, long-acting reversible, or hormonal method	9% (14/156)
	+ Pill + withdrawal	1.2% (2/156)		
	+ Pill	1.2% (2/156)		
	+ Other unique combinations	4.9% (7/156)^d^		
**SDM**				
	**1. Reported Current Use of SDM Plus Other Method**	**Weighted % Among Those Reporting SDM (unweighted n/N)**	**2. Classification Using “Most Effective Method” Approach**	**3. Among Women Who Reported SDM^a^ Weighted % (Unweighted n/N)**
A. Relies primarily on SDM	+ No other method	57.3% (39/68)	An SDM user	72% (49/68)
	+ Withdrawal	11.9% (8/68)		
	+ N Tablet	2.9% (2/68)		
B. May rely primarily on SDM and periodic use of EC and/or condoms^b^	+ Male condoms	8.6% (5/68)	A male condom or EC user	28% (19/68)
	+ Male condoms + EC + withdrawal	5.2% (3/68)		
	+ Male condoms + withdrawal	2.9% (2/68)		
	+ EC	2.9% (2/68)		
	+ Other unique combinations	8.4% (7/68)^e^		
C. Uses SDM but relies primarily on a modern method			A user of a permanent, long-acting reversible, or hormonal method	0% (0/68)

Abbreviations: EC, emergency contraception; SDM, Standard Days Method.

a Who would be classified as per label in column 2, if using the “most effective method” classification approach.

b 4 women reported using both rhythm and SDM, so they may rely primarily on either FABM. In DHS they would be classified as an SDM user.

c Rhythm plus male condoms and/or EC, and potentially one or more of the following: withdrawal, washing, other traditional method, or SDM.

d Rhythm plus 2 or more of the following: implants, 3 monthly injectables, pill, male condoms, LAM, EC, withdrawal, washing.

e SDM plus male condoms and/or EC, and potentially one or more of the following: rhythm, withdrawal, N Tablet.

We assumed that respondents in categories A and B (Table 2) should be counted as FABM users, suggesting that at least 18.1% of contraceptors in Ghana relied primarily on an FABM, whether modern (SDM: 6%) or traditional (rhythm: 12.4%) (Table 3). In other words, taking multiple method use (i.e., among women using an FABM plus (fe)male condoms and/or EC) into account increased the percentage of women who likely relied on an FABM from 13.5% (95% confidence interval [CI]=10.9, 16.7, and composed of 9.2% rhythm users and 4.3% SDM users) to 18.1% (95% CI=14.9, 21.8).

**TABLE 3.** Prevalence of FABMs by Estimation Method and Population Subgroup

	Rhythm % (95% CI)		SDM % (95% CI)		Either^b^ % (95% CI)	
	“Most Effective Method” Estimation Approach	Our Estimation Approach^a^	“Most Effective Method” Estimation Approach	Our Estimation Approach^a^	“Most Effective Method” Estimation Approach	Our Estimation Approach^a^
All women	2.4 (1.7, 3.3)	3.0 (2.2, 4.0)	1.1 (0.6, 1.9)	1.4% (0.9, 2.3)	3.5 (2.8, 4.4)	4.3 (3.4, 5.5)
Currently married women	3.2 (2.4, 4.4)	3.6% (2.6, 4.8)	1.7 (1.0, 2.8)	2.0 (1.2, 3.3)	4.9 (3.9, 6.2)	5.6 (4.3, 7.0)
Sexually active (within last30 days) unmarried women	3.6 (1.7, 7.1)	6.8 (3.9, 11.4)	1.4 (0.5, 4.0)	1.6 (0.6, 4.0)	5.0 (2.8, 8.6)	8.2 (5.2, 12.6)
Contraceptors	9.2 (6.9, 12.3)	12.4 (9.5, 15.9)	4.3 (2.5, 7.1)	6.0 (3.7, 9.4)	13.5 (10.9, 16.7)	18.1 (14.9, 21.8)

Abbreviations: CI, confidence interval; FABM, fertility awareness-based method; SDM, Standard Days Method.

a Incorporating information on multiple method use. This approach assumes that women in Categories A and B in Table 2 rely primarily on an FABM and may routinely or occasionally use condoms and/or EC during days they believe to be fertile.

b The “either” category does not always equal the sum of the “rhythm” and “SDM” category due to a small number of women who reported both methods.

### Characteristics Associated With FABM Use vs. Hormonal Contraception/IUD Use

In bivariate analyses, factors associated with approximately twice the odds of FABM use relative to hormonal contraception/IUD use included: living in the Middle or Central zone (vs. Northern zone), having never been (vs. currently being) married or cohabitating, feeling that avoiding pregnancy now was not at all (vs. very) important, identifying with any Christian religion (vs. no religion), and correctly identifying the approximate fertile time (vs. not knowing or correctly identifying it) (Table 4). Living in an urban (vs. rural) area and being in the richest 40% (vs. poorest 60%) of the population were associated with approximately 3 times the odds. Having attended secondary school (vs. no school) was associated with over 4 times the odds of FABM use (vs. hormonal contraception/IUD use). Each additional child born was associated with 20% lower odds of FABM use. Age was not significantly associated with FABM use.

**TABLE 4.** Odds Ratio Associated With Use of an FABM Relative to Use of Hormonal Contraception or an IUD

	Use of an FABM(Relative to Use of HormonalContraception/IUD)			
	Unadjusted Model^a^		Adjusted Model^±^	
	OR	95% CI	OR	95% CI
Zone (ref :Northern)				
Middle	1.9^b^	1.0, 3.5	1.5	0.7, 3.4
Central	2.0^b^	1.1, 3.8	1.1	0.4, 2.8
Residence (ref: rural)				
Urban	2.7^c^	1.5, 4.7	1.2	0.6, 2.4
Age (continuous)	1.0	0.9, 1.0	1.1^c^	1.0, 1.1
Union/marital status (ref: currently married/cohabitating)				
Formerly married or cohabitating	0.7	0.3, 1.5	0.6	0.2, 1.3
Never married or cohabitating	2.3^d^	1.3, 4.0	1.5	0.8, 2.9
Parity (continuous)	0.8^c^	0.7, 0.9	0.7^c^	0.6, 0.8
Education (ref: none)				
Attended primary or middle	1.7	1.0, 3.0	1.4	0.8, 2.5
Attended secondary	4.4^c^	2.3, 8.6	2.1^b^	1.1, 4.1
Religion (ref: no religion)				
Any Christian	2.0^b^	1.1, 4.0	1.1	0.6, 2.3
Muslim	1.5	0.7, 3.3	1.2	0.4, 3.3
Traditional religion/other	0.3	0.1, 1.1	0.3	0.1, 1.2
Wealth (ref: poorest 60%)				
Richest 40%	3.0^c^	2.0, 4.5	1.7^b^	1.0, 2.7
Importance of avoiding pregnancy now (ref: very important)				
Not at all important	1.8^b^	1.0, 3.2	1.9	0.9, 3.7
Somewhat important	1.3	0.6, 2.7	1.3	0.6, 3.2
Correct knowledge of approximate fertile time (ref: incorrect knowledge)				
Correct	1.9^d^	1.2, 3.1	1.5	0.9, 2.6

Abbreviations: CI, confidence interval; FABM, fertility awareness-based method; IUD, intrauterine device; OR, odds ratio.

a Model includes only contracepting women who rely or may rely primarily on an FABM (n=206), as well as users of hormonal contraception or an IUD (n=786).

± Adjusted for all other variables shown.

b *P*<.05.

c *P*<.001.

d *P*<.01.

In multivariable analysis, factors significantly associated with FABM use (vs. hormonal contraception/IUD use) included: age (each year was associated with an increase in odds of FABM use by 10%), parity (each child was associated with a decrease in odds of FABM use by 30%), and being richer or having attended secondary school or higher, each of which was associated with approximately double the odds of FABM use (Table 4). Feeling that avoiding pregnancy was not at all important and correct knowledge of the approximate fertile time were associated with an elevated odds ratio in bivariate analysis but did not reach statistical significance at *P*≤.05 in multivariable analysis.

Interestingly, among contraceptors, 34% of those not using an FABM and 50% of FABM users correctly identified the approximate fertile time (i.e., “halfway between 2 periods”) (*P*-value for chi^2^ = 0.01, Supplement B). It is also worth noting that most (80%) FABM users felt it was “very important” for them to avoid pregnancy now (Supplement B).

### How Women Report Using the Rhythm Method

The vast majority (85%) of rhythm users noted that they identify their fertile and nonfertile days by counting days of a menstrual cycle using a calendar (Supplement C) (participants could report more than 1 response). We did not obtain more detailed information on which rules, specifically, were used to determine fertile days using a calendar. About 1 in 6 rhythm users (17%) used an unspecified approach to determine her fertile window, and 9% did so by observing changes in cervical mucus. Alternative approaches were reported by 5% or less of rhythm users: using a mobile phone app (5%), observing the position or texture of their cervix (3%), recording daily temperature (1%), or using CycleBeads to determine her fertile days (0%).

Among rhythm users, 12% believed there was no chance of getting pregnant over the course of a year while using the method, and 64% reported believing that there was a ≥50% chance.

Nearly three-quarters (72%) of rhythm users had heard of the SDM. Less than one-quarter had heard of ways to make FABMs easier to use, such as CycleBeads (22%) or the CycleBeads mobile app (17%), other mobile FABM apps (23%), or other simple-to-use FABMs like TwoDay method (22%).

Nearly all rhythm users (92%) expressed interest in learning how to make the rhythm method more effective at pregnancy prevention. Among women who wanted to make their rhythm use more effective (and were not already using the given approach mentioned), a majority would be willing to learn about approaches facilitated by a phone (82%) or to record their daily temperature (76%). Slightly over half (53%) expressed a willingness to collect their cervical mucus daily or to observe the texture of their cervix by inserting their fingers into their vagina daily.

Only half of rhythm users knew where to go for advice on using rhythm effectively. Among those who knew, most (53%) would go to a family planning service provider. However, only 17% of rhythm users had ever discussed using the rhythm method with a health professional.

### How Women Report Using FABMs (Rhythm or SDM) Overall

When FABM users were asked how they avoided pregnancy on days identified as fertile (and permitted to list as many responses as desired), 74% reported avoiding intercourse (Supplement D). The next most common answer (19%) was withdrawal, followed by using condoms or another barrier method (16% among rhythm users, and 13% among SDM users), and EC (12% among rhythm users and 11% among SDM users).

The majority (64%) of FABM users who abstained from sex during the fertile window reported that it was “very easy” to get their partner to abstain on these days, with another 22% reporting that it was somewhat easy. Only 14% reporting that this was somewhat or very hard. Among FABM users who reported using withdrawal during fertile days, 68% said very easy, 10% somewhat easy, and 22% somewhat or very hard. We asked a similar question about condom use, but an ODK programming error precluded calculating estimates for this question.

When FABM users were asked whether menstruation affects sexual activity with their partner, 76% said “yes, we generally avoid sex when I am menstruating,” but 20% stated, “no, because we don't have sex regularly.” Only 4% responded, “no, we generally have sex when I am menstruating.”

## DISCUSSION

We posit that at least 18% of contracepting women in Ghana likely relied primarily on an FABM for pregnancy prevention. Had we relied on the most effective method reported, as is standard procedure, the estimate would be 13.5% (9.2% rhythm, 4.3% SDM). Our 18% estimate likely represents a lower bound, because, like the 2014 Ghana DHS^47^ and 2017 Ghana Maternal Health Survey,^48^ we used a filtering question.^†^ However, unlike those surveys, among women who acknowledged ever or currently doing something or using a method to prevent pregnancy, our survey asked about all methods. Also, unlike those surveys, we assessed patterns of multiple method use to ascertain likely FABM users.

We posit that at least 18% of contracepting women in Ghana likely relied primarily on an FABM for pregnancy prevention.

Our rhythm prevalence estimates (based on most effective method) were slightly lower than those from the 2017 Ghana Maternal Health Survey for all women, currently married women, and sexually active unmarried women (Supplement A). This may be because SDM users may have reported themselves as rhythm users in that survey; combining our rhythm and SDM estimates results in estimates similar to estimates for rhythm in that survey. Overall, based on our estimate that 18% of contracepting Ghanaian women likely rely primarily on an FABM, we estimate that a minimum of 343,890 Ghanaian women (of an estimated 1,899,943 Ghanaian female contraceptors) currently rely primarily on an FABM^49^; a larger number than those using oral contraceptive pills or using male condoms, and nearly as many as those using 3-monthly injectables.

Among FABM users, more than half (57%) reported currently using only their FABM, while the remainder also reported current use of condoms, withdrawal, EC, and/or other methods. Prior studies in Ghana suggest that such contraceptive “mosaics” of methods that are viewed as natural are common. These methods are believed to help protect the regular flow of menses from bleeding changes induced by other methods^50^ as bleeding changes are perceived as linked with infertility.^7^^,^^51^^–^^53^ These contraceptive patterns have implications for how FABM prevalence is calculated in large-scale surveys and merits re-examination of the use of terms like “periodic abstinence” and the definition of “rhythm” currently used in DHS and other survey questions. For example, the Ghana 2017 Maternal Health Survey describes rhythm as “to avoid pregnancy, women do not have sexual intercourse on the days of the month they think they can get pregnant”; this definition inaccurately implies that rhythm is always practiced with abstinence. We elaborate upon this concern in Supplement E, where we advocate that the term “periodic abstinence” be replaced with a less assumption-laden term, such as “fertility awareness-based method.” Similarly, we describe how language used to describe FABMs to respondents should be revised to avoid the assumption of abstinence during the fertile time.

Contraceptors who reported currently using FABMs (versus IUDs or hormonal methods) appeared to have several relatively advantaged characteristics: after controlling for multiple sociodemographic factors, they are more likely to be older, richer, more educated, and to have fewer children. This corroborates other findings from Ghana, in which contraceptors who were older, urban, more educated, and had fewer children were more likely to choose a traditional instead of modern method,^12^ though these patterns do not necessarily hold in other countries.^11^ Use of FABMs by relatively advantaged women may reflect distinct preferences for these methods, as identified in other Ghanaian studies^10^^,^^13^ and elsewhere^8^^,^^9^ or may reflect that aspects of FABMs may be less accessible to more disadvantaged women (e.g., power differentials negating an ability to negotiate the timing and circumstances of sexual activity with a partner, which women in our sample did not report particular difficulty in doing). Though only borderline statistically significant in our multivariable model, correct knowledge of the approximate fertile time did appear to be higher among likely FABM users, which has been observed previously in Ghana,^48^ yet only 50% of FABM users correctly identified the approximate fertile time, indicating substantial room for improvement among individuals relying upon this information to prevent pregnancy.

The majority (85%) of rhythm users reported identifying fertile and nonfertile days using a calendar, though it remains unclear exactly what rules they used to determine fertile and infertile days. Many were likely not using the formal rules of the rhythm method and may have simply avoided unprotected sex on days when they believed—possibly inaccurately—that pregnancy was possible.^16^ In Supplement E, we elaborate upon impreciseness in current use of the term “rhythm,” which has come to be used as a generic word for a diverse array of nonformalized practices,^54^ perhaps better described as “informal rhythm.” Furthermore, less than a quarter of rhythm users had heard of ways to make FABM use easier, and few reported using tools like CycleBeads or apps indicated for pregnancy prevention. Yet, nearly all rhythm users expressed interest in learning how to make use of their method more effective, and a substantial proportion of rhythm users expressed willingness to collect additional biomarkers (i.e., temperature, cervical mucus, cervical position) or to use tools such as mobile phone apps. Given that only 17% of rhythm users had ever discussed use of the rhythm method with a health professional, there appears to be a substantial missed opportunity to assist rhythm users in identifying ways to improve the effectiveness of their chosen contraceptive method.

Given that only 17% of rhythm users had ever discussed using the method with a health professional, there appears to be a substantial missed opportunity to assist users in identifying ways to improve the effectiveness of their chosen contraceptive method.

This missed opportunity has also been reflected in prior work in Ghana. For example, in a small (n=85) survey in central Ghana conducted in 2010 on contraceptive self-care options, only 36% of respondents indicated interest in self-administered injections, but 75% indicated wanting to learn more about FABMs.^55^ In a monitoring study of a free FABM app, 60.8% of Ghanaian women who downloaded the app for the purpose of pregnancy prevention were not using a method of contraception in the prior 3 months, and 23% noted that it was the first method they had ever used,^26^ suggesting that FABMs may attract new users. Researchers have recommended that family planning programs in Ghana consider the promotion of modern FABMs along with other modern methods,^55^^,^^56^ which would require health professionals (particularly community health nurses and midwives who commonly offer and provide family planning methods in Ghana) to be trained to offer FABMs. Furthermore, certain FABMs (e.g., CycleBeads) require commodities, but to the best of our knowledge, nationally representative, facility-based, reproductive health commodities and service surveys in Ghana funded by United Nations Population Fund have not included FABM-related commodities when assessing the availability of modern methods at service delivery points.

Strengths of this study include the use of a nationally representative sample of Ghanaian women and the incorporation of probing on the ever or current use of all methods of contraception. This builds upon an approach used by Rossier et al. in Burkina Faso, wherein investigators probed on all traditional methods.^33^ Finally, as far as we are aware, this is one of the first studies in a low- or middle-income country to ask detailed questions on FABM use, eliciting information that could be used to inform better outreach to and counseling for rhythm method users, who appear to be interested in understanding how to improve the effectiveness of their chosen contraceptive method.

### Limitations

Our study has multiple limitations, key among them being that our prevalence estimates suffer from some of the same methodological flaws as other nationally representative studies in Ghana, given that we used a filtering question in asking about method use, which may lead to underreporting of FABMs and other methods. We believe this would have been more impactful in soliciting “ever” use (vs. current use). If a participant acknowledged having ever used *any* method, interviewers would eventually ask about all methods, priming the respondent (before answering the current method question), that rhythm and SDM “count” as contraceptive methods. In addition, we did not specifically ask about the use of FABMs other than rhythm or SDM in our survey, which could potentially mean that users of methods such as TwoDay, symptothermal, or others are undercounted; however, we expect this to be highly unlikely, particularly given the responses to questions on knowledge of other FABMs. One of our questions on rhythm suffered from the same definitional errors as seen in other large-scale survey questions on rhythm. Specifically, one of our questions asked, “You noted that you use the rhythm method, or avoiding sex on days when you are more likely to get pregnant. Can you please state all of the ways in which you identify your fertile and non-fertile days?” The implicit assumption that rhythm users abstain from sexual activity on days they believe they are more likely to get pregnant should be discontinued in survey research on FABMs, as described above and elaborated upon in Supplement E. We do not know whether Ghanaian women who refer to their practice as “counting days” (as identified in other studies^34^) would label themselves as a rhythm user, an SDM user, a nonuser, or something else. Supplement E also highlights that the way in which users of specific FABMs (such as SDM, symptothermal method, etc.) are classified in DHS may depend upon whether the survey specifically asks about these methods or not; a point which researchers should be cognizant of when analyzing survey data.

Future surveys might more deeply investigate the use of menstrual trackers and how they are perceived, as well as examine whether variables not available in our data (i.e., distance to health facility) may be associated with FABM use. Finally, as noted previously,^46^ our approach to FABM prevalence estimation assumes that women reporting multiple contraceptive methods use them in conjunction with one another (rather than that, for example, a user was switching permanently from an FABM to condom use during the month of interview). Another complexity of understanding patterns of multiple method use is that our labeling of individuals being “primarily” FABM users is somewhat subjective; such users may perceive their “primary” method to be condoms or EC, for example, even if the FABM is being “used” throughout the cycle and condoms or EC are being used episodically during periods believed to be infertile.

## CONCLUSION

Our study indicates that a nontrivial proportion of reproductive age Ghanaian contracepting women (18%) are using a traditional or modern FABM (rhythm or SDM); more than those who report using oral contraceptive pills and nearly as many as report using 3-monthly injectables. Findings reiterate that standard approaches to measuring these methods underestimate their use, but additional survey methodological changes are required to further improve the accuracy of FABM use estimates. Results showed that more advantaged women were more likely to be using an FABM, suggesting that FABM use among this population may be a preference and not simply a lack of access to alternative options. Understanding these choices can help to support client-centered contraceptive counseling and programs for women and couples interested in using these methods. As side effects and health concerns are increasingly the main reasons for contraceptive nonuse in low- and middle-income countries, FABMs may offer an approach to pregnancy prevention to women whose contraceptive needs are not met by other options.^12^ Family planning programs should work to respectfully address these needs as part of a commitment to reproductive autonomy and choice.

## Supplementary Material

20-00601-Polis-Supplement.pdf

## Footnotes

* For comparability to other existing estimates, we also calculated contraceptive prevalence among all women, married women, and sexually active unmarried women (Supplement A).

† As described in the methods section, the filtering question excluded women who do not perceive themselves as “doing anything or using any method to delay or avoid getting pregnant.”

## Notes

**Peer Reviewed**

**First published online:** June 17, 2021.
