# Supplementary material for: Use of Fertility Awareness-Based Methods for Pregnancy Prevention Among Ghanaian Women: A Nationally Representative Cross-Sectional Survey
Source: Glob Health Sci Pract. 2021 Jun 30;9(2):318–31. doi: 10.9745/GHSP-D-20-00601 (PMC8324203; doi:10.9745/GHSP-D-20-00601)
Supplement: 20-00601-Polis-Supplement.pdf [file 20-00601-Polis-Supplement.pdf]

Supplement to: Polis CB, Otupiri E, Bell SO, Larsen-Reindorf R. Use of fertility awareness-based methods for pregnancy prevention among Ghanaian women: a nationally representative cross-sectional survey. *Glob Health Sci Pract*. 2021;9(2). https://doi.org/10.9745/GHSP-D-20-00601

Supplement A

**Current use of rhythm as most effective method**			
	**Ghana Demographic and Health Survey 2014**	**Ghana Maternal Health Survey 2017**	**Current survey 2018**
**All women**	3.1%	3.8%	2.4%
**Currently married women**	3.2%	4.2%	3.2%
**Sexually active (within last 30 days) unmarried women**	7.7%	5.1%	3.6%
**Current use of SDM as most effective method**			
	**Ghana Demographic and Health Survey 2014**	**Ghana Maternal Health Survey 2017**	**Current survey 2018**
**All women**	n/a	n/a	1.1%
**Currently married women**	n/a	n/a	1.7%
**Sexually active (within last 30 days) unmarried women**	n/a	n/a	1.4%

Supplement to: Polis CB, Otupiri E, Bell SO, Larsen-Reindorf R. Use of fertility awareness-based methods for pregnancy prevention among Ghanaian women: a nationally representative cross-sectional survey. *Glob Health Sci Pract*. 2021;9(2). https://doi.org/10.9745/GHSP-D-20-00601

Supplement B. FABM users and HC/IUD users by sociodemographic characteristics

	FABM users %	HC/IUD users %	Chi^2
**Zone**			0.23
Northern	7	12	
Middle	51	49	
Central	43	38	
**Residence**			<0.01
Urban	72	48	
Rural	28	52	
**Union/marital status**			0.01
Currently married/cohabitating	73	80	
Formerly married or cohabitating	6	9	
Never married or cohabitating	21	10	
**Education**			<0.001
None	6	13	
Attended primary or middle	53	67	
Attended secondary	41	20	
**Religion**			<0.001
No religion	4	7	
Any Christian	86	74	
Muslim	9	10	
Traditional religion/other	1	9	
**Wealth**			<0.001
Poorest 60%	37	64	
Richest 40%	63	36	
**Importance of avoiding pregnancy now**			0.10
Not at all important	13	8	
Somewhat important	7	6	
Very important	80	87	
**Correct knowledge of approximate fertile time**			<0.01
Correct	50	34	
Incorrect	50	66	
			T-test
**Age** (mean)	31.3	31.3	0.99
**Parity** (mean)	2.1	3.1	<0.001

Supplement to: Polis CB, Otupiri E, Bell SO, Larsen-Reindorf R. Use of fertility awareness-based methods for pregnancy prevention among Ghanaian women: a nationally representative cross-sectional survey. *Glob Health Sci Pract*. 2021;9(2). https://doi.org/10.9745/GHSP-D-20-00601

Supplement C. How women report using the rhythm method

**When using the rhythm method, how do you identify your fertile and non-fertile days?**	
Count days of menstrual cycle using a calendar	85%
Other (unspecified)	17%
Observe my cervical mucus	9%
Count days of menstrual cycle using a mobile phone app	5%
Observe the position or texture of my cervix	3%
Record my daily temperature	1%
Count days of my menstrual cycle using CycleBeads (SDM)	0%
**For a typical woman who uses the rhythm method to avoid pregnancy for one year, how likely do you think it is that she will become pregnant when she did not want to, on a scale from 0 to 10, with 0 being not at all likely to get pregnant and 10 being definitely will get pregnant?**	
0 (no chance of pregnancy in a year)	12%
1-4 (10-40% chance of pregnancy in a year)	21%
5 (50% chance of pregnancy in a year)	20%
6-9 (60-90% chance of pregnancy in a year)	34%
10 (100% chance of pregnancy in a year)	10%
**Have you ever heard of:**	
The Standard Days Method, in which women count days of their menstrual cycle and avoid unprotected intercourse on days 8-19 of their cycle?	72%
CycleBeads, a color-coded string of beads that helps women count days of her menstrual cycle and identify fertile and non-fertile days	22%
CycleBeads mobile app, which helps a woman count days of her menstrual cycle to identify fertile & non-fertile days	17%
Any other mobile phone app which helps a woman practice a fertility awareness based method of preventing pregnancy	23%
The TwoDay method of family planning, which relies upon a woman observing her daily cervical secretions to identify fertile and nonfertile days	22%
**Interested in learning about ways to make your use of the rhythm method more effective at preventing pregnancy**	92%
**(Among women who wanted to learn ways to make rhythm more effective and were not already using the given approach mentioned), would you be willing to:**	
Learn about natural methods of family planning facilitated by use of a phone?	82%
Take and record your daily temperature	76%
Collect and observe your cervical mucus on a daily basis	53%
Observe the daily texture of cervix by inserting your fingers into your vagina to feel your cervix	53%
**Do you know where to go for advice on using the rhythm method effectively?**	50%
**Where would you go if you wanted advice on using the rhythm method more effectively?**	
Family planning service provider	53%
Medical provider	18%
Midwife	17%
Community health worker	17%
Friend	16%
Internet	12%
Other (unspecified)	6%
Pharmacy	5%
Church	3%

Traditional healer	1%
**Have you ever discussed using the rhythm method with a health professional?**	17%

Supplement to: Polis CB, Otupiri E, Bell SO, Larsen-Reindorf R. Use of fertility awareness-based methods for pregnancy prevention among Ghanaian women: a nationally representative cross-sectional survey. *Glob Health Sci Pract*. 2021;9(2). https://doi.org/10.9745/GHSP-D-20-00601

Supplement D. How women report using FABMs overall

**When using this method, what do you do to avoid pregnancy on days you identify as fertile?** (multiple responses permitted)	**Rhythm (among 138 self-identified users, weighted)**	**SDM (among 68 self-identified users, weighted)**
Avoid intercourse	74%	74%
Use withdrawal	19%	19%
Use condoms or another barrier method	16%	13%
Use EC	12%	11%
Other	7%	3%
Use N Tablet	1%	6%
Nothing	<1%	0%
	**Rhythm or SDM (among 154 self-identified users, weighted)**	
**Among women who abstain on fertile days: how easy or difficult is it to get your partner to abstain on fertile days?**		
Very easy	64%	
Somewhat easy	22%	
Somewhat hard	6%	
Very hard	8%	
**Among women who use withdrawal on fertile days: how easy or difficult is it to get your partner to use withdrawal on fertile days?**		
Very easy	68%	
Somewhat easy	10%	
Somewhat hard	16%	
Very hard	6%	
**When you are menstruating, does this affect sexual activity with your partner?**		
Yes, we generally avoid sex when I am menstruating	76%	
No, we generally have sex with I am menstruating	4%	
No, because we don't have sex regularly	20%	

Note: The survey included a question "Among women who use condoms or another barrier method: how easy or difficult is it to get your partner to use condoms or another barrier method on your fertile days?" Due to a technical issue in data collection, estimates from this question are unfortunately unavailable.

Supplement E. Challenges in terminology/definitions for FABMs in Demographic and Health Surveys:

1. Inaccuracy in the label "periodic abstinence", and in the definitions used for periodic abstinence, rhythm, and Standard Days Method
2. Labeling changes (and conflation) over time for periodic abstinence and rhythm
3. Classification as a "periodic abstinence and/or rhythm" user depends on whether other methods (i.e., Standard Days Method, mucus method, etc.) were asked about

1. Inaccuracy in the term "periodic abstinence", and in the definitions used for periodic abstinence, rhythm, and Standard Days Method

Issue: The term "periodic abstinence" inherently assumes that users remain abstinent during days identified by the method as fertile. However, while some users of fertility awareness based methods (such as rhythm or SDM) abstain on days considered fertile, others may use other contraceptive methods (i.e., barriers, emergency contraception, withdrawal, and/or other methods) to avoid pregnancy during days identified as fertile, without periodically abstaining.

The definitions of "periodic abstinence" and/or "rhythm" have changed over time in the contraceptive knowledge section of DHS surveys (**Supplement Table E1**), but all definitions include the embedded assumption that sexual intercourse is avoided on days women are most likely to get pregnant.

Similarly, the Standard Days Method is defined as either "abstaining or using a condom on days identified as fertile". It should be clarified whether women using SDM rules and using withdrawal, EC, or non-condom barrier methods on fertile days are to be classified as SDM users or not.

Implications: Literal interpretation of the term "periodic abstinence" excludes FABM users who remain sexually active during the fertile window. Although women define for themselves whether they are a user of a particular method or not, they may be influenced by descriptions used by interviewers in the contraceptive knowledge section of the survey (which are used if a respondent indicates not having heard of the method). Given the descriptions used for rhythm and SDM, users of those methods may not recognize themselves as using those methods. Other studies (Fabic and Choi, *Studies in Family Planning*, 2013) have demonstrated improved reporting on lactational amenorrhea method when the description of this method was included in the contraceptive knowledge section.

Suggestion: The term "periodic abstinence" is unhelpful as an umbrella term, and should be replaced with "fertility awareness-based method" (unless being used specifically to describe FABM users who always abstain during the fertile time). The descriptions used for rhythm should be revised to avoid this issue, and it should be considered whether the

definition for Standard Days Method definition should be updated to clarify how people who use SDM with withdrawal, emergency contraception, or non-condom barrier methods during days identified as fertile should be classified.

2. **Labeling changes (and conflation) over time for periodic abstinence and rhythm**

   Issue: Phase 1 DHS model questionnaires (1984-1989) asked about "periodic abstinence" and did not mention "rhythm". Phases 2-4 (1988-2003) mentioned both terms, and used them interchangeably. Phases 5-8 (2003-present) asks only about "rhythm method" (**Supplement Table E1**).

   Implication: Some authors use the terms periodic abstinence and rhythm interchangeably. The rhythm method has a formal set of rules (though not all women who report using rhythm likely follow these rules), while periodic abstinence was likely intended to be used as an umbrella term (akin to "fertility awareness-based methods"). In practice, most "periodic abstinence" or "rhythm" users in DHS surveys to date are likely using a non-formal method that reflects colloquial understandings of "rhythm".

   Suggestion: Recent model surveys ask about "rhythm", and no longer use the term "periodic abstinence" – researchers should also discontinue general use of the term periodic abstinence. Greater precision would also be helpful when researchers and programmers use the word "rhythm", which can mean different things to different people. Specifically, some people use it to indicate implementation of the formal rules of the calendar-rhythm method (which involves complex calculations requiring details on the length of a person's last 6 menstrual cycles), while others interpret it to mean something less formal.

3. **Classification as a periodic abstinence/rhythm user depends on whether other methods (i.e., Standard Days Method, mucus method, etc.) were asked about**

   Issue: Only some DHS surveys have included Standard Days Method or Cycle Beads as a separate response option. In Phase 7 (2013-present), DHS model questionnaires began including Standard Days Method, but not all countries adhere to the model questionnaire. Some other specific FABMs (e.g., "mucus method", "symptothermal method", and others) have, less commonly, been included in some country- and year-specific DHS surveys.

   Implication: Respondents classified as a periodic abstinence/rhythm user may vary, depending on how respondents refer to their method and what prompts (for alternate FABMs) they do or do not hear during the survey. In countries where respondents were asked about specific other FABMs (e.g., Standard Days Method, TwoDay Method), self-identified users of those other FABMs should, in theory, be identified as users of that specific FABM (and thus excluded from the periodic abstinence/rhythm group).

However, the category of periodic abstinence/rhythm may include some women using other specific FABMs who were not identified as such through the survey.

Suggestion: Researchers should be cognizant of what categories are included in surveys they analyze, and describe potential implications of inclusion or exclusion of those categories on results.

Supplement to: Polis CB, Otupiri E, Bell SO, Larsen-Reindorf R. Use of fertility awareness-based methods for pregnancy prevention among Ghanaian women: a nationally representative cross-sectional survey. *Glob Health Sci Pract*. 2021;9(2). https://doi.org/10.9745/GHSP-D-20-00601

Supplement Table E1. Terms and definitions related to fertility awareness-based methods in model DHS questionnaires over time

	Did model questionnaire ask about periodic abstinence, rhythm, or both?	How was this label defined?	Did model questionnaire ask specifically about Standard Days Method?	How was Standard Days Method defined?
Phase 1 (1984-1989)	Periodic abstinence	Couples can avoid having sexual intercourse on certain days of the month when the woman is more likely to become pregnant	No	--
Phase 2 (1988-1993)	Rhythm, periodic abstinence	*Same as Phase 1*	No	--
Phase 3 (1992-1997)	*Same as Phase 2*	Every month that a woman is sexually active she can avoid having sexual intercourse on the days of the month she is most likely to get pregnant.	No	--
Phase 4 (1997-2003)	Rhythm or periodic abstinence	Every month that a woman is sexually active she can avoid pregnancy by not having sexual intercourse on the days of the month she is most likely to get pregnant.	No	--
Phase 5 (2003-2008)	Rhythm method	*Same as Phase 4*	No	--
Phase 6 (2008-2013)	*Same as Phase 5*	To avoid pregnancy, women do not have sexual intercourse on the days of the month they think they can get pregnant.	No	--
Phase 7 (2013-2018)	*Same as Phase 5*	*Same as Phase 6*	Yes	A woman uses a string of colored beads to know the days she can get pregnant. On the days she can get pregnant, she uses a condom or does not have sexual intercourse
Phase 8 (2018-2023)	*Same as Phase 5*	*Same as Phase 6*	Yes	*Same as above*
